# Active Learning for Node Classification: An Evaluation

**DOI:** 10.3390/e22101164

**Published:** 2020-10-16

**Authors:** Kaushalya Madhawa, Tsuyoshi Murata

**Affiliations:** Department of Computer Science, Tokyo Institute of Technology, Tokyo 152-8552, Japan; murata@c.titech.ac.jp

**Keywords:** machine learning, graph neural networks, node classification, active learning, graph representation learning

## Abstract

Current breakthroughs in the field of machine learning are fueled by the deployment of deep neural network models. Deep neural networks models are notorious for their dependence on large amounts of labeled data for training them. Active learning is being used as a solution to train classification models with less labeled instances by selecting only the most informative instances for labeling. This is especially important when the labeled data are scarce or the labeling process is expensive. In this paper, we study the application of active learning on attributed graphs. In this setting, the data instances are represented as nodes of an attributed graph. Graph neural networks achieve the current state-of-the-art classification performance on attributed graphs. The performance of graph neural networks relies on the careful tuning of their hyperparameters, usually performed using a validation set, an additional set of labeled instances. In label scarce problems, it is realistic to use all labeled instances for training the model. In this setting, we perform a fair comparison of the existing active learning algorithms proposed for graph neural networks as well as other data types such as images and text. With empirical results, we demonstrate that state-of-the-art active learning algorithms designed for other data types do not perform well on graph-structured data. We study the problem within the framework of the exploration-vs.-exploitation trade-off and propose a new count-based exploration term. With empirical evidence on multiple benchmark graphs, we highlight the importance of complementing uncertainty-based active learning models with an exploration term.

## 1. Introduction

Supervised learning is an important technique used to train machine learning models that are deployed in multiple real-world applications [1]. In a supervised classification problem, data instances with ground truth labels are used for training a model that can predict the labels of unseen data instances. Therefore, the performance of a supervised learning model depends on the quality and quantity of training data, often requiring a huge labeling effort. Usually, the labeling of data instances is done by humans. Labeling large amounts of data leads to a huge cost in both time and money. The labeling cost is significantly high when the labeling task needs to be done by domain experts. For example, potential tumors in medical images can be labeled only by qualified doctors [2,3].

With ever-increasing amounts of data, active learning (AL) is gaining the attention of researchers as well as practitioners as a way to reduce the effort spent on labeling data instances. Usually, a fraction of data instances are selected randomly and their labels are queried from an oracle (e.g., human labelers). This set of labeled instances are used for training the classifier. This process is known as *passive learning* [4] as the training data is selected at the beginning of the training process and it is assumed to stay fixed. An alternative approach is to iteratively select a small set of training instances, retrieve their labels, and update the training set. Then, the classification model is retrained using the acquired labeled instances and this process is repeated until a good level of performance (e.g., accuracy) is achieved. This process is known as *active learning* [5]. The objective of AL can be expressed as acquiring instances that maximize model performance. An *acquisition function* evaluates the informativeness of each unlabeled instance and selects the most informative ones. As quantifying the informativeness of an instance is not straightforward, a multitude of approaches have been proposed in AL literature [5]. For example, selecting the instance the model is most uncertain about is a commonly used acquisition function [6].

In this paper, we study the problem of applying AL for classifying nodes of an attributed graph (The term “network” is used as an alternative term in the literature. We use the term graph since the usage of the term network can be confused with neural networks in this paper.). This task is known as node classification. Reducing the number of labeled nodes required in node classification can benefit a variety of practical applications such as in recommender systems [7,8] and text classification [9] by selecting only the most informative nodes for labeling. Parisot et al. [3] demonstrated the importance of representing the associations between brain scan images of different subjects as a graph for the task of predicting if a subject has Alzheimer’s disease. Features extracted from images are represented as node attributes. This is an example for a node classification problem where labeling is expensive as labeling a brain scan image is time-consuming and it can only be done by medical experts.

Node classification is an important task in learning from relational data. The objective of this problem is to predict the labels of unlabeled nodes given a partially labeled graph. Different approaches have been used for node classification including iterative classification algorithm (ICA) [10], label propagation [11], and Gaussian random fields (GRF) [12]. Approaching node classification as a semisupervised problem has contributed to state-of-the-art in classification performance [13,14,15]. In a semisupervised learning problem, the learning algorithm can utilize the features of all data instances including the unlabeled ones. Only the labels of unlabeled instances are not known. Semisupervised learning is a technique that utilizes unlabeled data to improve the label efficiency. Combining AL with semisupervised learning can increase the label efficiency further [16]. Graph neural network (GNN) models have achieved state-of-the-art performance in node classification [17].

Similar to other neural network-based models, GNN models are sensitive to the choice of hyperparameters. The common hyperparameters of a GNN model are learning rate, number of hidden layers, and the size of hidden units of each hidden layer. Unlike model parameters, the hyperparameters are not directly optimized to improve the model performance. Finding the most suitable set of values for hyperparameters is known as hyperparameter tuning. It is usually performed based on the performance of the model on a separate held-out labeled set known as the validation set. It is possible to leave a fraction of labeled data as the validation set when labeled data is abundant. However, in a label scarce setting, it is realistic to use all the available labeled instances for training the model. Therefore, we further reduce the size of the labeled set by not using a validation set and using fixed standard values for hyperparameters.

With the recent popularity of GNNs, several surveys on GNNs have been done [17,18,19]. These works provide a comprehensive overview of recent developments in graph representation learning and its applications. Surveys on AL research have been done separately [20,21]. However, as far as the authors know, a survey and a systematic comparison of existing AL approaches for the task of node classification have not been done yet. Moreover, only a handful of graph datasets are used for benchmarking such models. Most of the benchmark graphs are similar as they come from the same domain. In this paper, we study commonly used AL acquisition functions on the problem of node classification using a multitude of graph datasets belonging to different domains. As shown in previous work [22], the performance of AL algorithms is not consistent across different datasets.

Our main contributions are

1. we discuss the importance of performing AL experiments in a more realistic setting where an additional labeled dataset is not used for hyperparameter tuning;
2. we perform a thorough evaluation of existing AL algorithms on the task of node classification of attributed graphs in a more realistic setting; and 
3. with empirical evidence on an extensive set of graphs with different characteristics, we highlight that graph properties should be considered in selecting an AL approach.

## 2. Background

### 2.1. Node Classification

Node classification plays an important part in learning problems when the data is represented as a graph. A graph *G* consists of *V* nodes and *E* edges connecting pairs of nodes. Edges of a graph can be directional as well. However, we limit our study to undirected graphs. Node classification is widely used in practical applications such as recommender systems [8,23], applied chemistry [24], and social network analysis [25]. In a node classification problem, an attributed graph G=(V,E) with *N* nodes is given as an adjacency matrix $A\in R^{N\times N}$ and a node attribute matrix $X\in R^{N\times F}$. Here, *F* is the number of attributes. An element $a_{ij}\in A$ represents the edge weight between two nodes $v_{i}$ and $v_{j}$. If there is no edge connecting $v_{i}$ and $v_{j}$, $a_{ij}=0$. If the graph is undirected, the adjacency matrix *A* is symmetric. The degree matrix *D* is a diagonal matrix defined as $D=\{d_{1},\cdots ,d_{N}\}$, where each diagonal element $d_{i}$ is the row-sum of the adjacency matrix such that $d_{i}=\sum_{j=1}^{N}a_{ij}$. Each node $v_{i}$ has a real-valued feature vector $x_{i}\in R^{N\times F}$ and $v_{i}$ belongs to one of the *C* class labels.

The objective of this problem is to predict the labels of unlabeled nodes $V_{U}$ given a small set of labels $V_{L}$. Earlier attempts for solving this problem relied on classifiers based on the assumption that nodes connected by an edge are likely to share the same label [26,27]. A major weakness of such classifiers is that this assumption restricts the modeling capacity and the node attributes are not used in the learning process. The use of node attributes of an attributed graph significantly improves the classification performance.

### 2.2. Graph Neural Networks (GNNs)

A GNN is a neural network architecture specifically designed for learning with attributed graphs. GNN models [14,28,29] achieve state-of-the-art performance on the node classification problem providing a significant improvement over previously used embedding algorithms [30,31]. What sets GNNs apart from previous models is their ability to jointly model both structural information and node attributes. In principle, all GNN models consist of a message passing scheme that propagates feature information of a node to its neighbors. Most GNN architectures use a learnable parameter matrix for projecting features to a different feature space. Usually, two or more of such layers are used along with a nonlinear function (e.g., ReLU). Let *G* be an undirected attributed graph represented by an adjacency matrix *A* and a feature matrix *X*. By adding self-loops to the adjacency matrix we get $\tilde{A}=A+I$ and its degree matrix $\tilde{D}=D+I$. Using this notation, the graph convolutional network (GCN) model [14] can be expressed as

(1) $${\tilde{H}}^{\left(k\right)}={\tilde{D}}^{-1/2}\tilde{A}{\tilde{D}}^{-1/2}H^{\left(k-1\right)},$$

where ${\tilde{D}}^{-1/2}\tilde{A}{\tilde{D}}^{-1/2}$ is the normalized adjacency matrix. Then, the hidden representation of a layer $H^{\left(k\right)}$ is obtained by multiplying the feature matrix ${\tilde{H}}^{\left(k\right)}$ with a parameter matrix θ and applying an activation function σ as

(2) $$H^{\left(k\right)}=\sigma \left({\tilde{H}}^{\left(k\right)}\theta^{\left(k\right)}\right).$$

With normalized adjacency matrix $\hat{A}={\tilde{D}}^{-1/2}\tilde{A}{\tilde{D}}^{-1/2}$ a two-layer GCN model [14] can be expressed as

(3) $$Y_{\mathrm{GCN}}=\mathrm{softmax}\left(\hat{A}\;\mathrm{ReLU}\left(\hat{A}X\theta^{\left(0\right)}\right)\theta^{\left(1\right)}\right),$$

where *X* is the node attribute matrix and $\theta^{\left(0\right)}$ and $\theta^{\left(1\right)}$ are the parameter matrices of two neural layers. The softmax function defined as $\mathrm{softmax}(x)=exp\left(x\right)/\sum_{c=1}^{C}exp\left(x_{c}\right)$ normalizes the output of the classifier across all classes. Rectified linear unit (ReLU) is a commonly used activation function where ReLU(x)=max(0,x).

Wu et al. [29] showed that a simplified GNN model named SGC can achieve competitive performance on most attributed graphs at a significantly lower computational cost. They obtained this model by removing hidden layers and nonlinear activation functions in the GCN model. This model can be written as

(4) $$Y_{\mathrm{SGC}}=\mathrm{softmax}\left({\hat{A}}^{k}X\theta \right),$$

where $A^{k}$ is the *k*th power of the adjacency matrix *A*. The parameter *k* determines the number of hops the feature vectors are propagated to. This approach is similar to propagating node attributes over the *k*-hop neighborhood and then performing logistic regression. Using a 2-hop neighborhood (k=2) often results in good performance.

### 2.3. Active Learning

In this paper, we consider the pool-based AL setting [5]. In a pool-based AL problem, the labeled dataset L is much smaller compared to a large pool of unlabeled items U. We can acquire the label of any unlabeled item by querying an oracle (e.g., a human annotator) at a uniform cost per item. Suppose we are given a query budget *K*, such that we are allowed to query labels of a maximum of *K* unlabeled items. We use the notation $f_{\theta }$ to denote a classification model with trainable parameters θ. The probability of an instance *q* belonging to class *c* predicted by this model is written as $P_{\theta }\left({\hat{y}}_{q}=c|x,D_{L}\right)$. We calculate this likelihood as

(5) $$P_{\theta }\left({\hat{y}}_{q}=c|x,D_{L}\right)=\mathrm{softmax}{\left(f_{\theta }\left(x_{q}\right)\right)}_{\left[q=c\right]}.$$

AL research has contributed to a multitude of approaches for training supervised learning models with less labeled data. We recommend the work in [5] as a detailed review of existing AL research. The objective of AL approaches is to select the most informative instance for labeling. This task is performed with the use of an acquisition function, where the acquisition function decides which unlabeled example should be labeled next. Existing acquisition functions can be grouped into a few general frameworks based on how they are formulated. In this section, we describe a few commonly used AL frameworks.

#### 2.3.1. Uncertainty Sampling

Uncertainty sampling [32] is one of the most widely used AL approaches. The active learner selects the instance for which the classifier predicts a label with the least certainty. The information entropy of the label predictions is usually used to quantify the uncertainty of the model for a given instance $x_{q}$ such that

(6) $$H\left(y_{q}|x,D_{L}\right)=-\sum_{c=1}^{C}P_{\theta }\left({\hat{y}}_{q}=c|x_{q},D_{L}\right)log\left(P_{\theta }\left({\hat{y}}_{q}=c|x_{q},D_{L}\right)\right).$$

The instance corresponding to the maximum entropy is selected for querying

(7) $$q^{*}=\underset{q}{arg max}H\left(y_{q}|x_{q},D_{L}\right).$$

The entropy computed over model predictions of a neural network does not correctly represent the model uncertainty for unseen instances. Even though Bayesian models are good at estimating the model uncertainty, Bayesian inference can be prohibitively time-consuming. Gal and Ghahramani [33] demonstrated that using dropout [34] at evaluation time is an approximation to a Bayesian neural network and this can be used to calculate the model uncertainty. Gal et al. [35] used this Bayesian approach to perform uncertainty sampling for active learning on image data with convolutional neural networks (CNN). Additionally, Gal et al. [35] performed a comparison of various acquisition functions proposed for quantifying the model uncertainty of CNN models. It is shown that uncertainty sampling is prone to select outliers [20].

Bayesian Active Learning by Disagreement (BALD) [6] is another uncertainty-based acquisition function used with Bayesian models. BALD algorithm selects the instance that maximizes the mutual information between the predictions and the model posterior. This can be written as

(8) $$q^{*}=\underset{q}{arg max}H\left(y_{q}|x_{q},D_{L}\right)-E_{\theta ∼p(\theta |D_{L})}\left[H(y_{q}|x_{q},\theta ,D_{L})\right].$$

The left side term of the Equation (8) is the entropy of the model prediction and the right side term is the expectation of the model prediction over the posterior of the model parameters. If the model is certain of its predictions for each draw of parameter values, the right side term becomes smaller. In this case the active learner selects the examples $x_{q}$ for which the model is most uncertain of its predictions (high $H(y_{q}|x_{q},D_{L})$), but the model is confident for individual parameter settings (low $E_{\theta ∼p(\theta |D_{L})}\left[H(y_{q}|x_{q},\theta ,D_{L})\right]$).

#### 2.3.2. Query by Committee (QBC)

Query by committee (QBC) [36] is a simple method that outperforms uncertainty sampling in many practical settings. This method maintains a committee of models trained on the same labeled dataset. Each model in the committee predicts the label of an unlabeled instance. The instance for which label predictions of the most number of committee members (models) disagrees is selected as the most informative instance. However, QBC is not a popular choice when AL is used with deep neural network (DNN) models since training a committee of DNN models is time-consuming.

#### 2.3.3. Expected Error Reduction (EER)

Expected Error Reduction (EER) [37] is an AL approach that directly calculates the expected generalization error of a model trained on labeled instances including unlabeled instances $L\cup (x_{q},y_{q})$. Then, the active learner queries the instance which minimizes the future generalization error. However, this approach involves the retraining of a model for each unlabeled instance $x_{q}$ with each label c∈C, making it one of the most time-consuming AL approaches. Therefore, the EER approach has been limited to simple classification algorithms such as Gaussian random fields (GRF) for which faster online retraining is possible.

## 3. Active Learning for Graph Classification Problems

Compared to application of AL on other types of data such as image and text data, only a limited number of AL models has been developed for graph data. Previous work on applying AL on graph data [38,39,40] is tightly coupled with earlier classification models such as Gaussian random fields, in which the features of nodes are not being used. Therefore, selecting query nodes uniformly in random coupled with a recent GNN model can easily outperform such AL models. AL models which utilize recent GNN architectures [41,42] are limited. Moreover, a comprehensive comparison of AL algorithms proposed for other domains of data has not been done yet.

In Table 1, we provide an extensive comparison of the literature on AL approaches proposed for node classification. We compare each work with respect to the following attributes.

- AL approach
- Classifier: Classification model used for predicting the label of a node
- Attributes: Whether the node classifier uses node attributes
- Adaptive: Whether the active learner is updated based on the newly labeled instances
- Labels: Whether the active learner uses node labels in making a decision

In addition to generic approaches proposed for AL, there have been a few works that are specifically designed for graph-structured data. These algorithms use graph-specific metrics for selecting nodes for labeling. In addition to the attributes of data instances, graph topology provides useful information. For example, the degree centrality of a node represents how a particular data instance is connected with others. Table 1 demonstrates that most of the previous AL approaches proposed for node classification do not use the node attribute information. Moreover, some works [40,43] ignore the label information as well.

### 3.1. Active Learning Framework

In this problem, we start with an extremely small set of labeled instances. We are given a query budget *K* such that we are allowed to query *K* number of nodes to retrieve their labels. In each acquisition step, we select a node and retrieve its label from an oracle (e.g., a human labeler). The GNN model is retrained using the training set including the newly labeled instance. We repeat this process *K* times. The basic framework is shown in Algorithm 1. Here, $f_{\theta }$ is any node classification algorithm with parameters θ and we can use different acquisition functions (e.g., uncertainty sampling or QBC) as *g*.
**Algorithm 1** Active learning for node classification.**Input:** Graph G=(A,X), Query budget *K*, Initial labels $Y_{L}$**Output:** An improved model $f_{\theta }$**for**i←1 to $n_{q}=K$ **do** Select the best unlabeled instance $q^{*}$ with an acquisition function *g* Retrieve its label $Y_{q^{*}}$ Update label set $Y_{L}\leftarrow Y_{L}\cup Y_{q^{*}}$ Retrain the model $\theta \leftarrow {arg min}_{\theta }l\left(f_{\theta }\left(G\right),Y_{L}\right)$**end for****Return θ**

### 3.2. The Importance of Exploration

After each acquisition step, the classifier is trained on a limited number of labeled instances, which in turn are selected by the active learner. Therefore, the selected labeled instances tend to bias towards instances evaluated to be “informative” by the active learner. Therefore, the distribution of labeled instances is often different from the true underlying distribution. The active learner cannot observe the consequences of selecting an instance which has lower “informativeness”. This leads the active learner to converge to policies that are not able to generalize for unlabeled data. This problem is amplified by the lack of hyperparameter tuning. A simple approach to overcome this limitation is to query a few instances in addition to the ones maximizing our selection criteria. This step is known as “exploration” while selecting the instance maximizing the criteria is “exploitation”. For example, if our criterion is model entropy, the exploration step involves acquiring labels of a few instances which do not have the maximum entropy. Intuitively, an active learner should perform more exploration initially, so it can have a better view of the true distribution of data.

This problem is known as the *exploration* vs. *exploitation trade-off* in sequential decision-making problems. Solving this trade-off requires the learner to acquire potentially suboptimal instances (i.e., exploration) in addition to the optimal ones. This problem is studied under the framework of multi-armed bandits (MAB) problems [46]. In a MAB problem, a set of actions are given and selecting an action results in observing a reward drawn from a distribution that is unknown to the learner. The problem is to select a sequence of actions that maximize the cumulative reward. A multitude of approaches is used in solving online learning problems modeled as MAB problems. ϵ-greedy, upper confidence bounds (UCB) [47], and Thompson sampling [48] are a few of the frequently used techniques.

We compare the performance of each active learner using two different exploration techniques: ϵ-greedy and count-based exploration.

#### 3.2.1. ϵ-Greedy

ϵ-greedy is used as the simplest method of introducing exploration into an MAB algorithm. In the case of AL, with probability ϵ the active learner randomly selects an unlabeled instance for querying its label. The most informative instance is selected by an acquisition function with probability (1−ϵ). A key problem with this approach is that, as each unlabeled instance is selected with uniform probability, some of the labeled instances can be wasteful. This phenomena is known as *undirected exploration* [49].

#### 3.2.2. Count-Based Exploration

In MAB problems, count-based exploration addresses the problem of undirected exploration by assigning a larger probability to actions that have been selected fewer times compared to the remaining actions. Based on the principle of optimism in the face of uncertainty, a count-based exploration algorithm computes an upper confidence bound (UCB) [47] and selects the action corresponding to the maximum UCB. We adopt the notion of count-based exploration as the number of labeled nodes in the neighborhood of an unlabeled node. We define the exploration term of an instance *i* as the logarithm of the number of unlabeled neighboring nodes of *i*. This term encourages the learner to sample nodes from neighborhoods with less number of labeled nodes. As this term and the informative metric used in the acquisition function (e.g., entropy) are on different scales, we normalize both of these quantities into [0,1] range and get $\phi_{\exp}\left(i\right)$ and $\phi_{\inf}\left(i\right)$, respectively. We linearly combine these normalized quantities to get the criterion for acquiring nodes as

(9) $$\phi \left(i\right)=\left(1-\gamma_{t}\right)\cdot \phi_{\inf}\left(i\right)+\gamma_{t}\cdot \phi_{\exp}\left(i\right),$$

where the exploration coefficient $\gamma_{t}$ is a hyperparameter that balances exploration and exploitation. Setting $\gamma_{t}$ to 0 corresponds to pure exploration disregarding the feedback of the classifier. On the other hand, $\gamma_{t}=1$ is equivalent to pure exploitation selecting a node based only on the uncertainty sampling (e.g., entropy).

## 4. Experiments

### 4.1. Data

We evaluate the performance of all algorithms on 11 real-world datasets belonging to different domains. as shown in Table 2. In Table 2, we list the datasets used in experiments with several graph properties. These datasets belong to different domains such as citation networks, product networks, co-author networks, biological networks, and social networks.

CiteSeer, PubMed, and CORA [50] are commonly used citation graphs. Each of these undirected graphs is made of documents as nodes and citations as edges between them. If one document cites another, they are linked by an edge. The bag-of-words features of the text content of a document correspond to the attributes of a node.

Co-author CS and Co-author Physics are co-authorship graphs constructed from Microsoft Academic Graph [51]. Authors are represented as nodes and two authors are linked by an edge if they have co-authored a paper. Node features correspond to the keywords of the papers authored by a particular author. An author’s most active field of study is used as the node label.

Amazon Computers is a subgraph of the Amazon co-purchase graph [52]. Products are represented as nodes, and two nodes are connected by an edge of those two products that are frequently bought together. Node attributes correspond to product reviews encoded as bag-of-words features. The product category is used as the node label.

The disease dataset [53] simulates the SIR disease propagation model [54] on a graph. The label of a node indicates whether a node is infected or not and the features indicate the susceptibility to the disease.

The Wiki-CS dataset [55] is a graph constructed from Wikipedia articles corresponding to computer science. A Wikipedia article is a node of this graph and two nodes are connected by an edge if one article has a hyperlink to the other. GloVe word embeddings [56] obtained from the text content of an article is used as the feature vector of the node corresponding to that article.

Each protein–protein interaction (PPI) graph represents physical contacts between proteins in a human tissue (brain, blood, and kidney) [57,58]. Unlike other datasets, in PPI graphs a protein (node) can have multiple functions as its label, making this a multi-label classification problem. Learning the protein function (cellular function from gene ontology) involves learning about node roles. Several properties of a protein such as positional gene sets, motif gene sets and immunological signatures are used as node attributes in a PPI graph.

Github is a social network dataset constructed from developer profiles on Github who have at least 10 public repositories [59]. Details of a developer such as location, employee, and starred repositories are represented as node attributes. Two nodes are linked by an edge if those two developers mutually follow each other on Github. The label of a node indicates whether a developer is primarily working on machine learning or web development projects.

From each dataset, we randomly select two nodes belonging to each label as the initial labeled set $V_{L}$. We use 5% of the rest of the unlabeled nodes as the test set. The set of remaining unlabeled nodes $V_{U}$ qualify to be queried. The size of the initial labeled set and its size as a fraction of the total nodes (labeling rate) are shown in Table 2.

#### Graph Properties

In some real-world graphs, such as social and communication networks, nodes tend to cluster together creating tightly knit groups of nodes. This phenomenon is known as clustering and the clustering coefficient [60] quantifies the amount of clustering present in a graph. The local clustering coefficient of a node *i* is calculated as

(10) $$C_{i}=\frac{\mathrm{number}\;\mathrm{of}\;\mathrm{triangles}\;\mathrm{connected}\;\mathrm{to}\;\mathrm{node}\;i}{\mathrm{number}\;\mathrm{of}\;\mathrm{triples}\;\mathrm{centered}\;\mathrm{around}\;\mathrm{node}\;i}.$$

Average clustering coefficient is calculated as the average of local clustering coefficients of all nodes of a graph.

In real-world graphs, nodes tend to connect with other nodes with similar properties. In network science literature this phenomenon is known as “assortative mixing” [61]. Assortativity coefficient quantifies the amount of assortative mixing present in a graph. Assortativity coefficient can be calculated with respect to any node attribute. We calculate the label assortativity ($r_{L}$) with

(11) $$r_{L}=\frac{\sum_{i}e_{ii}-\sum_{i}a_{i}b_{i}}{1-\sum_{i}a_{i}b_{i}},$$

where $e_{ij}$ denotes the fraction of edges connecting a node with label *i* with a node with label *j*. For multi-label graphs, we calculate label assortativity for each label separately and take the average. A higher label associativity indicates that a node tends to connect with another node with the same label. As shown in Table 2, citation and co-author graphs exhibit high assortativity. It is easier to predict labels in a graph exhibiting high assortativity since neighbors of a node tend to have the same label as the node. Many node classification models are based on this assumption. However, the PPI graphs show low assortativity indicating that nodes with the same label are not necessarily in the same neighborhood. This is due to the fact that the function of a protein (i.e., node) depends on the role of a node in that graph rather than its neighboring proteins (i.e., nodes). Using degree centrality as a node attribute degree assortativity $r_{D}$ of each node can be computed in a similar manner. Average degree assortativity of a graph indicates whether a high degree node prefers to connect with other high degree nodes.

### 4.2. Experimental Setup

#### 4.2.1. Node Classification Model

Recent studies demonstrated that GNN-based classifiers significantly outperform previous classifier algorithms such as GRFs. Therefore, we restrict our study of AL to GNN-based learning models. In our experiments, we consider two types of graph neural network architectures: GCN [14] and SGC [29]. SGC is a simplified GNN architecture that does not include a hidden layer and nonlinear activation functions. As the goal of AL is to reduce the number of labeled instances used for training, we do not use a separate validation set for fine-tuning the hyperparameters of a GNN model. In addition, it is shown that tuning hyperparameters while training a model with AL can lead to label inefficiency [62].

For all datasets, we use the default hyperparameters used in GNN literature (e.g., learning rate = 0.01). We use the following algorithms in our experiments.

- Random: Select an unlabeled node randomly,
- PageRank: Select the unlabeled node with the largest PageRank centrality,
- Degree: Select the unlabeled node with the largest degree centrality,
- Clustering coefficient: Select the unlabeled node with the largest clustering coefficient,
- Entropy: Calculate the entropy of predictions of the current model over unlabeled nodes and select the node corresponding to the largest entropy.,
- BALD [6,35]: Select the node which has the the largest mutual information value between predictions and model posterior, and
- AGE [41]: Select the node which maximizes a linear combination of three metrics: PageRank centrality, model entropy and information density.

Here, PageRank, degree, and clustering coefficient-based sampling do not use node attributes or the feedback from the classification model. On the other hand, entropy BALD are uncertainty-based acquisition functions that calculate an uncertainty metric using the performance of the classifier trained using the current training set. We acquire the label of an unlabeled node and retrain the GNN model by performing 50 steps of adam optimizer [63]. We perform 40 acquisition steps (query budget = 40) and repeat this process on 30 different randomly initialized training and test splits for each dataset. Test dataset is often unbalanced. Therefore, accuracy is not suitable to be used as the performance metric. We report the average F1 score (macro-averaged) over the test set in each experiment. F1-score is the harmonic mean of the precision and recall metrics. Macro-F1 score is calculated by first calculating F1-scores for each class separately and then taking the average of class-wise F1-scores.

#### 4.2.2. Packages and Hardware

We use the NetworkX library [64] for representing and processing graphs. We use the Pytorch [65] implementations of GCN [14] and SGC [29] node classification models. All experiments are run on a computer running Ubuntu 18.04 OS on an Intel(R) Core i9-7900X CPU @ 3.30GHz processor with 64GB memory and a NVIDIA GTX 1080-Ti GPU.

## 5. Results and Discussion

### 5.1. Performance Comparison of AL Approaches

**Figure 1:**
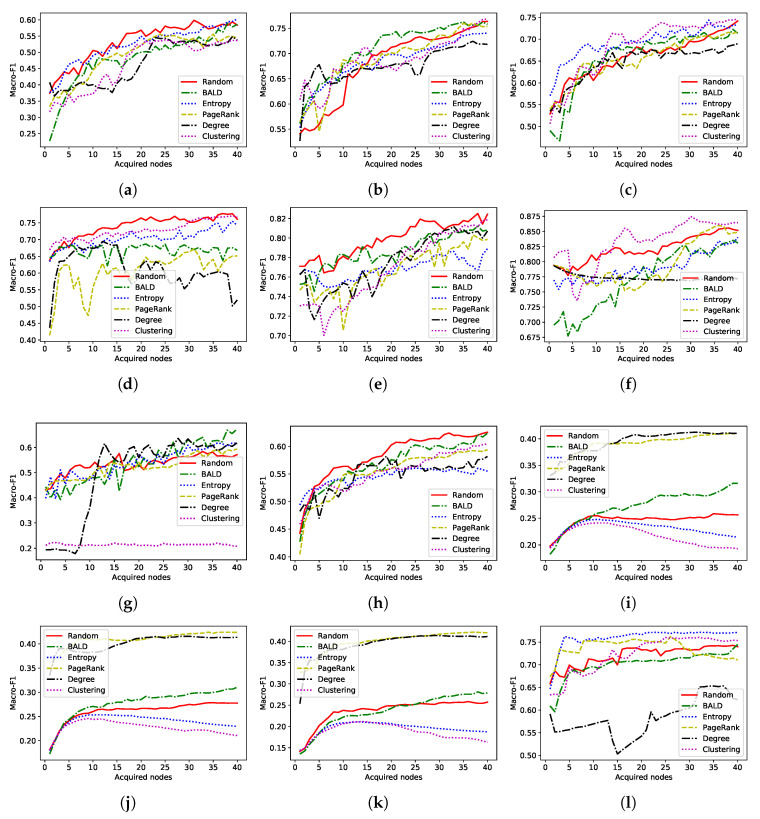
Macro-F1 score (test) of active learning algorithms with number of acquisitions. A two-layer graph convolutional network (GCN) is used as the graph neural network (GNN) model. (**a**) CiteSeer. (**b**) PubMed. (**c**) CORA. (**d**) Amazon Computers. (**e**) Co-author CS. (**f**) Co-author Physics. (**g**) Disease. (**h**) Wiki-CS. (**i**) PPI-Brain. (**j**) PPI-Blood. (**k**) PPI-Kidney. (**l**) Github.

**Figure 2:**
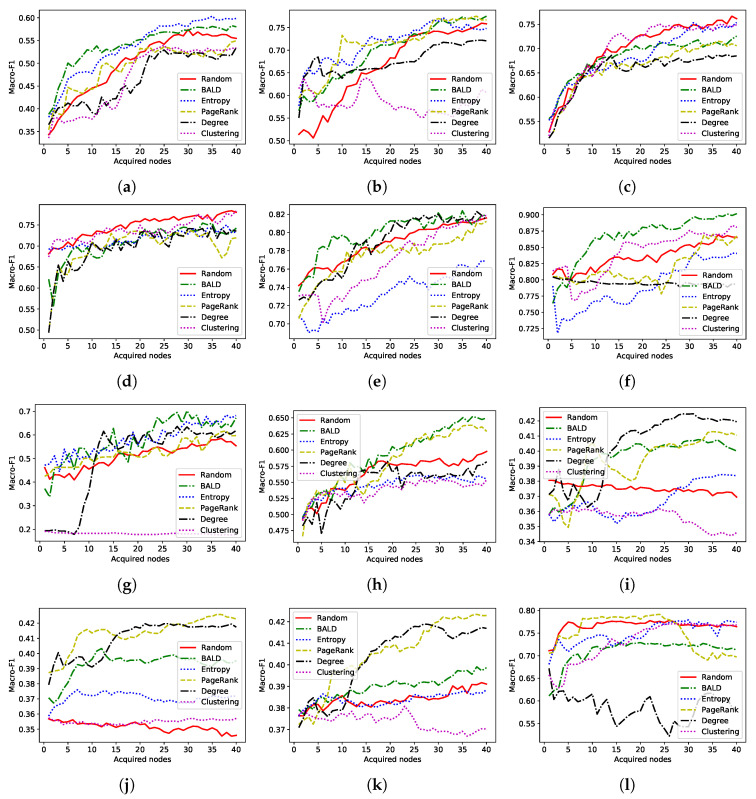
Macro-F1 score (test) of active learning algorithms with number of acquisitions. SGC model is used as the GNN model. (**a**) CiteSeer. (**b**) PubMed. (**c**) CORA. (**d**) Amazon Computers. (**e**) Co-author CS. (**f**) Co-author Physics. (**g**) Disease. (**h**) Wiki-CS. (**i**) PPI-Brain. (**j**) PPI-Blood. (**k**) PPI-Kidney. (**l**) Github.

In this section, we compare the performance of acquisition functions which use only a single type of approach. Figure 1 and Figure 2 show how the performance of the node classification model varies with the number of acquisitions.

As shown in previous works, AGE [41], the current state-of-the-art AL algorithm, performs well on citation networks (CiteSeer, CORA, and PubMed). However, the performance of this algorithm is suboptimal on other datasets such as Wiki-CS. The citation datasets possess similar characteristics. For example, average degree centrality of them is in the same range as shown in Table 2. Therefore, selecting AL algorithms based on their performance on a handful of graphs from the same domain may result in suboptimal algorithms.

### 5.2. Comparison of Exploration Strategies

In this experiment, we run uncertainty sampling algorithms: BALD and entropy with ϵ-greedy and count-based exploration terms. In the count-based exploration policy, we set the exploration coefficient β to 0.5. In Table 3 and Table 4, we present the performance of GCN and SGC classifiers when 40 nodes are acquired using each of the acquisition functions. Entropy-Count and BALD-Count correspond to max entropy sampling and BALD policy combined with count-based exploration term. The values in bold indicate that the performance of an algorithm is significantly better (at 5% significance level) than the rest of the algorithms on that dataset. We calculate the statistical significance between the performance of two algorithms using paired t-test. If no single algorithm is significantly better than the rest, all statistically significant values are marked in bold. We summarize the results in Table 5 and show the best performing AL algorithm along with the classifier. Uncertainty-based acquisition functions, when combined with the count-based exploration term (Entropy-Count and BALD-Count), achieve the best performance on average on four datasets. It highlights that encouraging the active learner to select nodes in less explored neighborhoods is effective than selecting a node in random as the exploration step (ϵ-greedy).

### 5.3. Running Time

Table 6 shows the execution time each algorithm spends to acquire a set of 40 unlabeled instances on average. AGE, the current state-of-the-art, is several magnitudes slower compared to the rest of the algorithms. The clustering step performed to compute the information gain is responsible for the additional time. The time complexity of this step grows $O(n^{2})$ with the number of vertices *n* of a graph making AGE not suitable for large attributed graphs. For example, the AGE algorithm is 80 times slower than random sampling for the Amazon Computers graph but achieves inferior performance. Additionally, the SGC model can be trained in a relatively less time compared to the GCN model and this difference is significant for larger graphs such as Wiki-CS and co-authorship graphs. However, in AL problems, the time spent for selecting an unlabeled example is a minor concern since the labeling time is more valued.

### 5.4. Discussion

As shown in Table 5, the performance of acquisition functions is diverse such that no single approach can be considered the best for all datasets. Sampling nodes based on graph properties leads to good performance depending on the graph structure. We make several key observations on how average clustering coefficient and label assortativity of a graph impact the performance of AL acquisition functions as following.

**Graphs with high level of clustering.** Amazon computers, co-authorship graphs, and Wiki-CS graphs have larger average clustering coefficients. For these datasets, sampling the node with the largest clustering coefficient outperforms sampling with other node centrality measures.

**Graphs with medium level of clustering.** CiteSeer, CORA, Github, and PPI graphs possess a medium level of average clustering in the range of 0.1 to 0.2. On CORA, CiteSeer, and Github datasets uncertainty-based acquisition functions and their variants obtain the best performance. However, the performance of PPI graphs is quite different since their label assortativity values are significantly low compared to all other datasets. 

**Graphs with low level of clustering.** Pubmed and the disease graphs have the lowest average clustering coefficients. In most cases, the use of clustering coefficient to select the nodes for querying lead to suboptimal results. However, sampling with clustering coefficient on PubMed dataset obtained good performance when the GCN model was used as the node classifier.

**Graphs with low label assortativity.** Out of all graph datasets, PPI graphs exhibit the lowest label assortativity. As most of the graphs used in node classification literature exhibit high label assortativity, commonly used node classification models are build on the assumption that neighbors of a node may have the same label. Therefore, such models are not confident in predicting the labels of unlabeled nodes, specially when the training data is scarce. On PPI graphs, we observe that performing AL by sampling the query nodes based on PageRank and degree centrality contributes to the best performing models. However, one limitation in calculating the label assortativity is that node labels need to be known beforehand. When we are given an unlabeled graph, one way to overcome this problem is we can use similar labeled graphs belonging to the same domain to approximate the label assortativity.

## 6. Conclusions

In this paper, we studied the application of the active learning framework as a method to make node classification on attributed graphs label efficient. We have performed an empirical evaluation of state-of-the-art active learning algorithms on the node classification task using twelve real-world attributed graphs belonging to different domains. In our experiments, we initiate the active learner with an extremely small number of labeled instances. Additionally, we assumed a more realistic setting in which the learner does not use a separate validation set. Our results highlight that no single acquisition function can be performs consistently well on all datasets and the performance of acquisition functions depend on graph properties. We further show that selecting an acquisition function based on the performance on a handful of attributed graphs with similar characteristics result in suboptimal algorithms. Notably, our results point that SGC, a simpler variant of GNN performs better and efficiently on most datasets compared to more complex GNN models.

A key takeaway of this research is that AL is beneficial in reducing the labeling cost of semisupervised node classification models and the choice of an AL acquisition function depends on the properties of the graph data at hand. Using an extensive set of graph datasets with a wide variety of characteristics, we showed that there is no single algorithm that works across different graph datasets possessing different graph properties. We further made the observation that using node PageRank and degree centrality of nodes achieve the best performance on graphs with low label assortativity.

Moreover, the current state-of-the-art active learning algorithm (AGE) [41] uses a combination of multiple acquisition functions and it is several magnitudes slower than all other acquisition functions that were used in this paper. Therefore, it is not suitable for large real-world attributed graphs. Lack of hyperparameter tuning and a minuscule number of training instances lead to classifiers that cannot generalize well for unlabeled data. We expressed this problem as balancing the exploration-vs.-exploitation trade-off and propose introducing an exploration term into acquisition functions. We evaluated the performance of two exploration terms using multiple real-world graph datasets. The introduction of this exploration term into existing uncertainty-based acquisition functions make their performance competitive with the current state-of-the-art AL algorithm for node classification on some datasets. As future work, we would like to explore how AL can be utilized for other graph-related learning tasks.

## Figures and Tables

**Table 1:** Summary of existing work for active node classification on attributed graphs. The work by Gadde et al. [43] does not use the labels of the nodes. Therefore, this method does not use a classifier. We use the following abbreviations in the table. LR—Logistic Regression, GRF—Gaussian Random Fields, LP—Label Propagation, SC—Spectral Clustering, NA—Not Applicable.

Work	AL Approach	Classifier	Attributes	Adaptive	Labels	Year
Zhu et al. [26]	EER	GRF	No	No	Yes	2003
Macskassy [44]	EER + Heuristics	GRF	No	Yes	Yes	2009
Bilgic et al. [39]	QBC	LR	No	Yes	Yes	2010
Gu and Han [38]	EER	LP	No	No	Yes	2012
Ji and Han [40]	Variation Minimization	GRF	No	No	No	2012
Ma et al. [45]	Uncertainty	GRF	No	No	Yes	2013
Gadde et al. [43]	SC	NA	No	No	No	2015
Cai et al. [41]	Uncertainty + Heuristics	GCN	Yes	Yes	Yes	2017

**Table 2:** Dataset statistics. Labeling rate as a percentage of total nodes is shown within brackets. Avg. deg.: Average degree, Avg. CC: Average clustering coefficient, $r_{D}$: Degree assortativity, $r_{L}$: Label assortativity.

Dataset	Nodes	Classes	Avg. Deg.	Avg. CC	$r_{D}$	$r_{L}$	Features	Labels (%)
CiteSeer	2110	6	2.84	0.17	0.007	0.67	3703	12 (0.56)
PubMed	19,717	3	6.34	0.06	−0.044	0.69	500	6 (0.03)
n CORA	2485	7	4.00	0.24	−0.071	0.76	1433	14 (0.56)
Amazon Comp.	13,752	10	36.74	0.35	−0.057	0.68	767	20 (0.14)
Co-author Phy	34,493	5	14.38	0.38	0.201	0.87	8415	10 (0.03)
Co-author CS	18,333	15	8.93	0.34	0.113	0.79	6805	30 (0.16)
Disease	1044	2	2.00	0.0	−0.544	0.68	1000	4 (0.38)
Wiki-CS	11,701	10	36.94	0.47	−0.065	0.58	300	20 (0.17)
PPI-Brain	3480	121	31.94	0.17	−0.064	0.09	50	35 (1.0)
PPI-Blood	3312	121	32.91	0.18	−0.061	0.09	50	33 (1.0)
PPI-Kidney	3284	121	31.70	0.18	−0.067	0.09	50	33 (1.0)
Github	37,700	2	15.33	0.17	−0.075	0.38	4005	4 (0.01)

**Table 3:** Average F1-score of different acquisition functions. Forty query instances are selected (average of 30 runs). Standard deviation is shown underneath the macro-averaged F1-score. Classifier: GCN. Rand—Random, Ent—Entropy, PR—PageRank, Deg—Degree, CC: Clustering coefficient.

Dataset	Rand	Ent	BALD	PR	Deg	CC	Ent ϵ-Greedy	BALD ϵ-Greedy	Ent Count	BALD Count	AGE
CiteSeer	58.4 ± 6.9	60.1 ± 8.1	58.6 ± 5.1	54.4 ± 3.4	53.8 ± 4.3	53.6 ± 7.0	59.4 ± 4.2	52.0 ± 5.7	60.3 ± 4.2	59.1 ± 4.7	**61.5** ± 3.7
CORA	74.1 ± 5.1	73.9 ± 6.6	71.4 ± 7.4	71.8 ± 5.1	70.2 ± 4.9	**74.5** ± 5.5	**75.1** ± 4.1	70.4 ± 6.4	73.3 ± 5.4	72.9 ± 3.6	**74.5** ± 7.7
PubMed	76.4 ± 4.0	74.1 ± 3.0	75.7 ± 3.8	75.3 ± 3.3	71.8 ± 3.7	76.8 ± 1.4	74.2 ± 4.2	74.1 ± 4.1	**77.3** ± 1.7	75.7 ± 4.4	74.5 ± 2.2
Coauthor CS	82.4 ± 2.3	78.9 ± 3.4	80.6 ± 3.6	79.7 ± 3.6	80.7 ± 2.5	81.9 ± 3.7	78.2 ± 4.4	81.2 ± 2.3	80.3 ± 4.9	82.1 ± 2.5	**83.9** ± 2.2
Coauthor Phy	85.2 ± 3.3	84.1 ± 3.2	83.8 ± 2.8	85.2 ± 1.8	77.1 ± 2.1	86.4 ± 2.9	85.5 ± 2.7	83.4 ± 3.5	86.9 ± 2.9	83.7 ± 2.8	**87.9** ± 2.6
Amazon Comp.	**76.1** ± 5.4	74.2 ± 3.4	66.8 ± 7.6	65.2 ± 8.1	60.2 ± 15.6	**76.7** ± 4.1	73.1 ± 6.0	70.8 ± 8.1	75.4 ± 3.8	73.3 ± 7.5	74.2 ± 5.9
Disease	57.1 ± 7.1	**67.1** ± 8.7	**67.2** ± 8.7	59.4 ± 8.8	53.2 ± 9.1	20.8 ± 5.1	61.0 ± 10.7	**66.5** ± 9.4	65.8 ± 9.2	**67.2** ± 7.2	63.3 ± 8.0
Wiki-CS	57.1 ± 7.1	55.0 ± 5.1	**62.4** ± 2.5	59.4 ± 3.1	58.2 ± 2.2	60.5 ± 3.7	61.0 ± 10.7	**63.3** ± 2.9	57.0 ± 3.3	**62.1** ± 3.7	57.7 ± 4.9
PPI Brain	25.6 ± 6.5	21.4 ± 6.3	31.6 ± 6.1	**41.1** ± 2.1	**41.0** ± 2.4	19.3 ± 6.6	22.3 ± 6.0	30.0 ± 8.9	22.2 ± 5.0	35.3 ± 6.1	22.3 ± 6.2
PPI Blood	27.7 ± 3.2	22.9 ± 5.7	31.0 ± 6.5	**42.4** ± 1.6	41.4 ± 1.9	21.0 ± 5.8	26.5 ± 4.9	36.9 ± 4.6	23.6 ± 5.4	37.4 ± 4.3	23.3 ± 5.6
PPI Kidney	25.7 ± 2.9	18.7 ± 6.8	27.9 ± 9.6	**42.1** ± 1.6	41.1 ± 2.2	16.3 ± 5.9	18.8 ± 7.0	33.5 ± 3.3	29.2 ± 1.7	37.6 ± 3.4	19.4 ± 4.8
Github	74.0 ± 8.0	**77.1** ± 1.9	74.5 ± 2.4	71.1 ± 2.9	62.3 ± 4.8	75.4 ± 1.8	**77.3** ± 1.6	74.4 ± 2.2	76.4 ± 2.2	73.8 ± 2.3	73.9 ± 2.1

**Table 4:** Average F1-score of different acquisition functions. Forty query instances are selected (average of 30 runs). Standard deviation is shown underneath the macro-averaged F1-score. Classifier: SGC. Rand - Random, Ent—Entropy, PR—PageRank, Deg—Degree, CC: Clustering coefficient.

Dataset	Rand	Ent	BALD	PR	Deg	CC	Ent ϵ-Greedy	BALD ϵ-Greedy	Ent Count	BALD Count	AGE
CiteSeer	55.5 ± 4.6	**59.9** ± 4.7	58.0 ± 4.0	55.0 ± 3.4	53.4 ± 5.3	53.4 ± 7.4	56.3 ± 5.6	56.0 ± 4.6	**60.0** ± 6.3	56.6 ± 4.8	**60.4** ± 5.6
CORA	**76.1** ± 3.7	75.4 ± 4.0	71.4 ± 2.3	71.4 ± 5.1	69.3 ± 3.4	74.9 ± 6.1	73.9 ± 6.1	73.8 ± 4.4	**76.7** ± 6.1	74.2 ± 3.1	74.7 ± 6.4
PubMed	75.8 ± 3.6	74.8 ± 2.3	77.5 ± 2.6	76.7 ± 2.5	72.3 ± 6.4	60.7 ± 7.9	75.3 ± 3.7	77.2 ± 2.4	76.6 ± 2.8	**78.0** ± 1.7	**77.7** ± 3.4
Coauthor CS	81.7 ± 2.9	76.8 ± 3.4	81.9 ± 3.9	81.3 ± 4.1	81.4 ± 4.0	81.9 ± 3.7	76.9 ± 4.1	82.6 ± 3.7	77.2 ± 4.7	**82.7** ± 4.8	**83.2** ± 2.9
Coauthor Phy	86.5 ± 3.3	84.1 ± 2.4	**90.2** ± 0.9	86.7 ± 2.9	79.3 ± 3.7	88.1 ± 2.7	84.1 ± 3.1	89.6 ± 2.6	87.5 ± 3.6	**90.4** ± 1.4	88.9 ± 2.1
Amazon Comp.	77.3 ± 4.1	73.4 ± 4.2	74.2 ± 5.3	71.9 ± 3.5	73.5 ± 6.1	**78.3** ± 3.6	75.8 ± 5.4	74.5 ± 6.7	74.3 ± 3.2	74.9 ± 5.3	75.6 ± 3.8
Disease	55.4 ± 8.7	**68.2** ± 6.1	**67.2** ± 7.1	59.7 9.5	58.5 ± 8.9	17.8 ± 4.5	63.4 ± 7.5	67.4 ± 8.5	67.1 ± 9.7	66.2 ± 8.4	66.4 ± 11.1
Wiki-CS	59.8 ± 6.3	55.5 ± 3.6	64.7 ± 4.0	62.9 ± 3.6	61.3 ± 3.1	55.4 ± 6.6	57.5 ± 5.3	63.8 ± 2.4	56.5 ± 5.8	**65.6** ± 3.1	50.4 ± 5.7
PPI Brain	36.9 ± 2.2	38.4 ± 2.4	40.0 ± 1.4	41.0 ± 1.4	**41.8** ± 1.2	34.6 ± 3.6	38.2 ± 2.0	40.3 ± 1.4	40.6 ± 1.0	**41.6** ± 1.2	33.2 ± 2.7
PPI Blood	34.6 ± 2.2	37.2 ± 3.7	39.5 ± 2.6	**42.3**± 2.3	41.7 ± 2.1	35.7 ± 1.7	37.0 ± 3.6	39.0 ± 2.9	39.8 ± 2.0	40.8 ± 2.1	39.4 ± 2.2
PPI Kidney	39.1 ± 1.8	38.8 ± 2.6	39.9 ± 1.4	**42.3** ± 1.8	41.7 ± 2.0	37.0 ± 1.4	40.0 ± 1.7	39.9 ± 1.4	40.4 ± 2.1	41.0 ± 1.8	41.0 ± 1.7
Github	76.4 ± 2.5	**77.4** ± 2.1	71.4 ± 2.5	69.7 ± 2.8	58.0 ± 5.6	76.8 ± 1.4	**77.4** ± 2.2	72.8 ± 1.5	75.8 ± 2.7	72.9 ± 1.5	73.3 ± 4.0

**Table 5:** The best performing model according to Table 3 and Table 4.

Data	Without Exploration			With Exploration		
	Macro-F1	Model	Classifier	Macro-F1	Model	Classifier
CiteSeer	61.5	AGE	GCN	61.5	AGE	GCN
CORA	76.1	Random	SGC	76.7	Entropy Count	SGC
PubMed	77.7	AGE	SGC	78.0	BALD Count	SGC
Coauthor CS	83.9	AGE	GCN	83.9	AGE	GCN
Coauthor Phy	90.2	BALD	SGC	90.4	BALD Count	SGC
Amazon Comp.	78.3	Clustering	SGC	78.3	Clustering	SGC
Disease	68.2	Entropy	SGC	68.2	Entropy	SGC
Wiki-CS	64.7	BALD	SGC	65.6	BALD Count	SGC
PPI Brain	41.8	Degree	SGC	41.8	Degree	SGC
PPI Blood	42.4	PageRank	GCN	42.4	PageRank	GCN
PPI Kidney	42.3	PageRank	SGC	42.3	PageRank	SGC
Github	77.4	Entropy	SGC	77.4	Entropy	SGC

**Table 6:** Running time (seconds): average execution time to acquire 40 unlabeled instances. We run all experiments on a single NVIDIA GTX 1080-Ti GPU. PR: PageRank, CC: Clustering coefficient.

Clf.	Dataset	Rand	Ent	PR	Deg	CC	AGE	BALD	ϵ-Greedy		Count	
									Ent	BALD	Ent	BALD
GCN	CiteSeer	4.2	4.8	4.8	4.7	4.9	4.8	4.8	4.8	4.8	4.8	4.8
	PubMed	6.9	7.6	25.4	7.3	32	1125.9	7.9	7.5	7.8	7.6	7.9
	CORA	4.2	4.5	4.6	4.4	14.5	26.8	4.5	4.5	4.5	4.5	4.5
	Coauthor CS	20.4	22.3	40.8	21.9	39.3	2154.2	23.7	22.3	23.6	22.4	23.6
	Coauthor Phy	46.1	50.5	116.4	48.5	98.6	2436.9	50.8	50.4	50.7	50.5	50.8
	Amazon Comp.	17.5	19.1	31.8	18.8	33.8	1688.9	19.2	19.1	19.1	19.1	19.2
	Disease	4.1	4.3	4.2	4.1	4.2	8.7	4.3	4.3	4.3	4.3	4.3
	Wiki-CS	15.3	16.6	30.0	28.3	33.0	410.8	16.7	16.6	16.6	16.7	16.7
	PPI Brain	8.3	8.9	11.5	10.2	10.9	133.3	9.0	8.4	8.6	8.4	8.7
	PPI Blood	7.9	8.2	10.4	9.4	9.9	130.2	8.4	8.2	8.4	8.3	8.5
	PPI Kidney	7.3	7.8	9.8	8.0	8.8	129.4	7.7	7.7	7.7	7.8	7.9
	Github	57.1	69.2	211.8	102.9	121.4	6810.0	72.1	69.6	71.1	70.5	73.2
SGC	CiteSeer	1.7	1.9	5.6	1.8	2.7	18.3	1.9	1.9	1.9	1.9	1.9
	PubMed	2.0	2.2	3.9	2.2	21.1	1229.2	2.2	2.2	2.2	2.2	2.2
	CORA	3.8	4.8	5.8	4.7	2.3	23.7	4.9	4.8	4.8	4.8	4.9
	Coauthor CS	16.8	19.8	33.2	19.3	37.9	2098.2	19.8	19.8	19.8	19.8	19.8
	Coauthor Phy	35.6	40.7	90.4	39.8	88.7	2232.3	40.8	40.4	40.5	40.7	40.7
	Amazon Comp.	12.2	14.7	17.2	16.9	17.1	1134.6	14.8	14.6	14.7	14.8	14.8
	Disease	1.4	1.4	1.5	1.4	1.4	6.0	1.4	1.4	1.4	1.4	1.4
	Wiki-CS	1.9	2.0	13.6	8.2	18.3	400.5	2.1	2.0	2.0	2.1	2.1
	PPI Brain	4.4	4.5	5.1	4.8	4.9	142.2	4.6	4.4	4.6	4.5	4.7
	PPI Blood	4.1	4.3	4.9	4.7	4.8	139.4	4.4	4.3	4.3	4.4	4.5
	PPI Kidney	3.9	4.1	4.4	4.3	4.5	135.6	4.1	4.1	4.1	4.1	4.2
	Github	22.3	24.5	166	78.3	106.2	4905.1	25.8	24.4	25.4	24.6	26.0

## Abbreviations

The following abbreviations are used in this manuscript.

DNN	Deep neural network
GCN	Graph convolutional network
GNN	Graph neural network
SGC	Simplified graph convolution
AL	Active learning
CNN	Convolutional neural network
BALD	Bayesian Active Learning by Disagreement
QBC	Query by committee
EER	Expected error reduction
GRF	Gaussian random fields
AGE	Active graph embedding
PR	PageRank
UCB	Upper confidence bound
